# Parenteral Nutrition-Induced Cholestasis in Neonates: Where Does the Problem Lie?

**DOI:** 10.1155/2013/163632

**Published:** 2013-11-14

**Authors:** Kheira Jolin-Dahel, Emanuela Ferretti, Carolina Montiveros, Renee Grenon, Nick Barrowman, Carolina Jimenez-Rivera

**Affiliations:** ^1^University of Ottawa, Ottawa, ON, Canada K1H 8L1; ^2^Department of Pediatrics, Children's Hospital of Eastern Ontario, 401 Smyth Road, Ottawa, ON, Canada K1H 8L1; ^3^Department of Statistics, Children's Hospital of Eastern Ontario, 401 Smyth Road, Ottawa, ON, Canada K1H 8L1

## Abstract

*Background*. Parenteral nutrition (PN) is an effective method of nourishing the neonate who is unable to receive full enteral feeds. Cholestasis can be a complication of PN and can lead to severe liver damage. *Aim*. We describe our patient population and determine risk factors for developing PN cholestasis. *Methods*. Retrospective chart review of newborns admitted from January 2006 to May 2011 to the Neonatal Intensive Care Unit at our institution and received PN >14 days. Cholestasis was defined as serum conjugated bilirubin >50 **μ**mol/L. *Results*. Eighty-seven newborns were included; 18 (20.7%) developed PN cholestasis. The most frequent surgical condition for both groups was gastroschisis (8/87; 9.2%). No significant differences were found between the cholestasis and control groups for the following parameters: birth weight, gestational age, intrauterine growth restriction, Apgar scores, and day of life at initiation of enteral feeds. Duration of PN in days and dosage of carbohydrates in g/kg/day were significantly higher in the cholestasis group than the control group. *Conclusion*. PN-related cholestasis presented in one-fifth of neonates receiving PN for more than two weeks. Longer duration of PN and higher dosage of carbohydrates were independent risk factors for the development of PN cholestasis in this population.

## 1. Introduction

The use of parenteral nutrition (PN) alone or in combination with enteral nutrition in neonates is effective in providing sufficient nutrients to maintain growth in the ill newborn infant. Cholestasis is a frequent complication of PN [1, 2]. The etiology of PN liver disease is unknown and likely multifactorial [3]. There have been multiple risk factors associated with PN-related cholestasis such as low birth weight [4], prematurity, duration of PN, sepsis, absence of enteral feeding, quality or quantity of amino acid intake [5, 6], male gender [7], mineral trace toxicity, toxicity from plant phytosterols [8], and perinatal depression or shock [6]. Intestinal resection and its complications have been also associated with the occurrence of PN-related cholestasis [2]. In some cases, progressive liver damage, liver failure, and death can occur [9]. 

Our aim was to describe the prevalence and risk factors for developing PN-related cholestasis in the neonatal period at the level III Neonatal Intensive Care Unit (NICU) at the Children's Hospital of Eastern Ontario.

## 2. Methods

We conducted a retrospective chart review of neonates admitted to the NICU at the Children's Hospital of Eastern Ontario who received PN for more than 14 days from January 1, 2006, to May 31, 2011. PN-related cholestasis was defined as an elevation of conjugated bilirubin greater than or equal to 50 *μ*mol/L subsequent to initiation of PN.

Clinical information included gestational age, birth weight, gender, Apgar score at 5 minutes less than 6, number of episodes of necrotizing enterocolitis and sepsis, gastrointestinal tract anomalies, bowel resection, day of life at initiation of PN, duration of PN, parenteral dosage in g/kg/d of protein, carbohydrates and lipids, vitamin and mineral trace element intake, and day of life (DOL) when enteral feeds were started.

Baseline laboratory investigation included alanine aminotransferase (ALT), aspartate aminotransferase (AST), alkaline phosphatase, gamma glutamyl transpeptidase (GGT), and bilirubin (conjugated and total) levels. Hepatic synthetic function included albumin and coagulation tests (international normalized ratio and partial thrombin time). Routine blood tests as per PN protocol were performed twice a week.

Patients who were found to have a conjugated bilirubin level above 50 *μ*mol/L were classified as the cholestasis group. Follow-up blood work was recorded at discharge, transfer, or resolution of PN-related cholestasis. Infectious causes of cholestasis including viral serology (Hep B sAg, Hep B sAb, Hep A IgM and IgG, Hep C serology, EBV, and toxoplasmosis) were included in our data files. Metabolic and endocrine disorders (such as *α* − 1 antitrypsin deficiency, and galactosemia, hypothyroidism) were ruled out as well as anatomical obstructive causes (e.g., biliary atresia) and Alagille syndrome.

Characteristics of PN solution (concentration of lipids, amino acids, vitamin, and mineral trace element) were recorded.

Between-group comparisons of categorical variables were performed using Fisher's exact test and Pearson's chi-squared test as appropriate. Between-group comparisons of continuous variables were performed using Mann-Whitney *U* test. Two-sided *P* values <0.05 were considered statistically significant. Unadjusted and adjusted associations between potential risk factors, like duration of TPN and dosage of carbohydrates, lipids, and amino acids, and cholestasis were examined using logistic regression. Odds ratios were obtained, together with 95% confidence intervals. To summarize the fitted risk, predicted probabilities of cholestasis were obtained at the midpoints of intervals of the risk factors that were found to be statistically significant in the multiple logistic regression model.

## 3. Results

There were 2,487 neonates admitted to the NICU during the study period. Eighty-seven newborns met the study criteria of receiving PN more than 14 days and were included in this study. The mean age of initiation of PN was 5 ± 9 d of life. Demographic distribution of patients between both groups was compared and no difference was found between the control and cholestasis groups, except for higher number of males in the control group. We also compared medical conditions distribution and incidence of sepsis and episodes of necrotizing enterocolitis (NEC) between both groups and found no statistical difference. The most common gastrointestinal abnormality present in both groups was gastroschisis (8/87; 9.2%). Patient characteristics are reported in Table 1. 

Eighteen (20.7%) out of 87 neonates had values of conjugated bilirubin ≥50 *μ*mol/L and were considered to have cholestasis; the remaining 69 served as the control group. Median DOL of bilirubin >50 *μ*mol/L was 32.4 ± 22 d; peak bilirubin level was 90 *μ*mol/L (IQR 68, 130 *μ*mol/L). Children with cholestasis also had elevation of liver enzymes with a mean ALT of 140 ± 195 U/L and AST of 215 ± 367 U/L. Other causes of cholestatic liver disease including infectious, metabolic, endocrine, obstructive, and syndromic causes were ruled out.

### 3.1. Parenteral Nutrition (PN)

**Figure 1:**
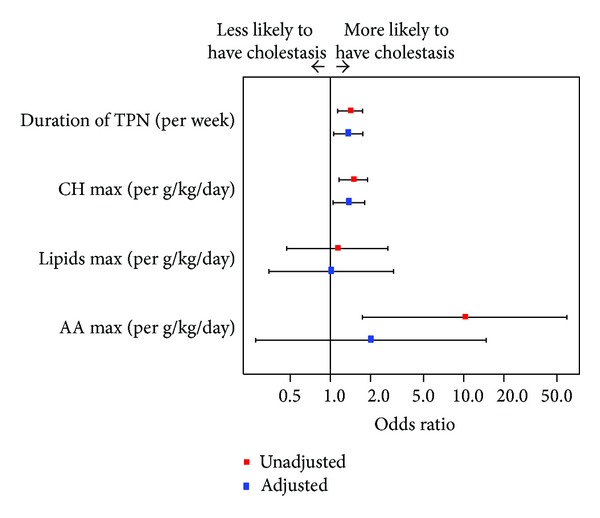
Unadjusted and adjusted odds ratios from logistic regression.

The duration of PN and dosage of each nutrient were compared between both groups. Neonates in the cholestasis group received PN for a longer period than the control group (39 d versus 20 d; *P* = 0.001). The dosage of carbohydrates in g/kg/day was significantly higher in the cholestasis group than in the control group (14.4 versus 12.5; *P* = 0.002). We found no significant differences in the dosage of amino acids and lipids for both groups. Dosage and duration of PN are reported in Table 2; unadjusted and adjusted odds ratios for cholestasis are reported in Figure 1. In the multiple logistic regression model, only duration of TPN (adjusted OR 1.36 per week, 95% CI 1.06–1.75) and dosage of carbohydrates (adjusted OR 1.38, 95% CI 1.05–1.81) were statistically significant.

Six neonates (6/18; 33.3%) in the cholestasis group and 1 (1/69; 1.4%) in the control group received Omegaven starting at mean day 21 into the course of receiving parenteral nutrition (range = 8–39 d). Characteristics of patients receiving Omegaven are shown in Table 3.

Table 4 describes the probability of developing cholestasis using a logistic regression model including duration of PN and dosage of carbohydrates. 

## 4. Discussion

Cholestasis is a commonly described complication of PN. Its etiology is not fully understood and is thought to be multifactorial [9]. Proposed mechanisms included altered bile salt metabolisms secondary to prematurity and toxic effect of PN components on the liver and gastrointestinal systems [10]. Aggravating factors such as sepsis and duration of bowel rest have also been described [11, 12]. 

### 4.1. Prematurity, Low Birth Weight, and Duration of PN

Our study did not reveal differences among premature and full-term newborns in the cholestatic and control groups. Prematurity, low birth weights, and duration of PN are often described as risk factors for developing PN-induced cholestasis [13, 14]. These risk factors are hard to separate because premature and low birth weight newborns will likely require PN for longer periods of time [15].

In our study, we did not find a higher proportion of intrauterine growth restriction (IUGR) in the cholestasis group. Baserga and Kelly [4, 15] compared the incidence of PN-induced cholestasis in extremely low birth weight infants who were either premature or had suffered from IUGR. They found that the IUGR group had a higher incidence of PN-induced cholestasis (56% versus 27%). Similiar studies have shown these findings and attributed them to metabolic and physiological changes to the hepatocytes secondary to uteroplacental insufficiency [15–17], specifically alteration in expression of glucose transporters [18] and higher susceptibility to infections [19]. Both of their groups had similar composition in PN nutrients. However, their IUGR group had a significantly longer duration of enteral rest which has also been identified as a risk factor for developing PN-induced cholestasis.

We identified, however, that the cholestasis group received PN for a longer period of time (39 versus 20 d; *P* = 0.003) compared to the control group. Our data suggests that the duration of PN itself is a risk factor for cholestasis similar to what is described in the literature [20].

### 4.2. Duration of Enteral Rest

When comparing the DOL at which enteral feeds were introduced and sustained, we did not observe any significant difference between the control and the cholestasis groups. Moreover, many patients (11 in the cholestasis and 51 in the control groups) were receiving at least some enteral stimulation concomitant to the administration of PN. These results support the hypothesis that the PN mixture causes toxicity, independently of whether there is enteral stimulation or not. However, with these results, we cannot exclude the possibility of length of enteral rest as an aggravating factor.

Lack of enteral stimulation has been suggested to be a risk factor for developing PN-induced cholestasis by various mechanisms [19, 21–24]. First, it is thought that lack of enteral stimulation reduces growth factors secretions that would normally promote enterocyte maturations; second, there is also a decrease in secretion of gut hormones such as cholecystokinin, hence promoting bile stasis and decreasing enterohepatic circulation [15]. Moreover, intestinal stasis leads to bacterial overgrowth. Studies have shown that endotoxins from gram-negative bacteria can inhibit bile secretions leading to cholestasis and that premature infants are more susceptible to these toxins [15, 25, 26].

### 4.3. Composition of PN Solution

#### 4.3.1. Amino Acids

There are numerous reports favoring the different components of PN as the main cause of its toxicity. Vileisis et al. [27] reported that patients receiving a higher dosage of amino acids in PN (high: 3.6 g/kg/day compared to low: 2.3 g/kg/day) developed cholestasis in a shorter period of time and the peak bilirubin level was higher. Our data analysis demonstrated no difference in the concentration of amino acids when comparing both groups. Within our cholestasis group, all patients had a similar amino acid dosage, as seen by the small standard deviation, despite the wide range in onset of cholestasis. It did not appear that amino acid dosage was a risk factor in our patient population in the multivariate analysis. 

#### 4.3.2. Carbohydrates

We did, however, find that our cholestasis group received a higher dosage of carbohydrates in their PN composition (14.4 versus 12 g/kg/d; *P* = 0.02), in agreement with previously published results identifying high carbohydrate content as a risk factor for developing PN cholestasis [27, 28].

Animal models have shown that high dextrose levels in PN solutions correlate with altered levels of insulin and glucagon in plasma as well as alteration in hepatocyte morphology, associated with increased periportal fatty infiltration [29]. In addition, it has been recommended that dextrose levels in the PN-solution should be administered at levels not greater than 7 g/kg/day [19]. 

#### 4.3.3. Lipids

In our study, we did not find any difference in the lipid dosage when comparing both groups, agreeing that lipid concentration is not a sole risk factor for developing cholestasis. It has been previously suggested that caloric excess caused by lipid overload could lead to steatosis and cholestasis [15, 27, 30, 31]. Specifically, it has been recommended that lipid concentration should not exceed 2.5 g/kg/day [32] and that dosage higher than 1 g/kg/day correlated with liver damage [33, 34]. Although excess lipid has been shown to accumulate in Kupffer cells [35, 36], there is no evidence that it directly causes cholestasis and if so, the mechanism is still not well understood [13, 27, 37–39]. 

#### 4.3.4. Omegaven

In our study, only six of 18 patients who developed PN-induced cholestasis received Omegaven. Unfortunately, this small number was not enough to generate meaningful statistical analysis. So far, studies demonstrated that patients who received Omegaven after developing PN-induced cholestasis have a higher rate of reversal when compared to patients who received soy-based emulsion [40]. Animal studies have proven that parenteral fish oil did not impair bile secretion and prevented steatosis [41, 42]. Soy-based fat emulsions contain phytosterols that have been recently identified as primary offending agents in PN-related cholestasis [15, 32, 43]. Fish oil, on the other hand, does not contain any phytosterols and contains omega-3 polyunsaturated fatty acids which have known anti-inflammatory properties [44–46].

In summary, PN-related cholestasis was not highly prevalent in our patient population, with only 20.7% compared to 24% and 35% reported by other authors [47, 48]. One of the limitations of this study is the small number of patients in the cholestasis group as well as patients receiving Omegaven, which did not allow for comparisons amongst the groups. However, we conclude that the onset of PN-induced cholestasis is likely multifactorial. We identified two risk factors by our data analysis: duration of PN and high levels of carbohydrates. We believe that our predicted probability of developing PN cholestasis (see Table 4) could serve as a guideline to adjust or modify these two factors to prevent the occurrence of this condition in the newborn.

## Figures and Tables

**Table 1:** Demographics of patients (exposed to PN for at least 2 weeks).

	Control (*N* = 69)	Cholestasis (*N* = 18)	*P* value
Gender, male (%)	37 (53.6)	15 (83.3)	0.023^¤^
Birth weight, grams, mean (SD)	1,781 (±1013)	1980 (±997)	0.340^¤^
Gestational age, *N* (%)			
<32 + 0/7 WGA	31 (44.9)	8 (44.4)	0.759^s^
32 + 0/7–36 + 6/7 WGA	18 (26)	4 (22.2)	
37 + 0/7–38 WGA	4 (5.8)	1 (5.6)	
>38 WGA	16 (23.2)	5 (27.8)	
Apgar < 6 at 5 min, *N* (%)	14 (20.3)	4 (23.5)	0.515^†^
IUGR, *N* (%)	10 (14.5)	2 (11.1)	1.00^s^
Number of episodes of sepsis, mean (SD)	0.78 (±0.9)	0.72 (±1.0)	0.645^¤^
Top 2 GI abnormality			
No. 1	Gastroschisis (6)	Gastroschisis (2)	NA
No. 2	Bowel perforation (5)	NA	NA

PN: parenteral nutrition, IUGR: intrauterine growth restriction, NEC: necrotizing enterocolitis, GI: gastrointestinal, WGA: weeks of gestational age, ^s^Fisher's exact test, ^¤^Mann-Whitney *U*, ^†^Pearson chi-squared test.

**Table 2:** Composition and duration of PN.

	Control (*N* = 69)	Cholestasis (*N* = 18)	*P* value
Duration of PN, days, median (IQR)	20 (15–31)	39 (24.2–53.0)	0.001^¤∗^
Introduction of enteral feeds DOL, median (IQR)	8.0 (3.3–19.8)	14.0 (5.5–36.5)	0.29^¤^
CH g/kg/day, median (IQR)	12.5 (10.0–13.2)	14.4 (12.5–16.2)	0.002^¤∗^
Lipids g/kg/day, median (IQR)	3.0 (3.0-3.0)	3.0 (3.0–3.4)	0.49^¤^
AA g/kg/day, median (IQR)	3.5 (3.0–3.5)	3.5 (3.5–4.0)	0.01^¤^

PN: parenteral nutrition, CH: carbohydrate, aa: amino acid, DOL: days of life. ^¤^Statistical analysis with the Mann-Whitney test. *P value is statistically significant.

**Table 3:** Patient characteristics within cholestasis group.

	Omegaven (*N* = 6)	No Omegaven (*N* = 12)
Gender, males (%)	6 (100)	9 (75)
Birth weight (g), Mean (SD)	1, 598 ± 614	2, 171 ± 1117
Preterm, *N* (%)	6 (100)	7 (58.3)
IUGR, *N* (%)	6 (100)	2 (16.7)
Apgar < 6, *N* (%)	6 (100)	4 (36.4)
DOL conj bili above 50 *μ*mol/L, mean (SD)	21.7 ± 12.69	37.8 ± 24.67
1st conj bili above 50 *μ*mol/L, mean (SD)	64.7 ± 15.4	67.4 ± 13.8
Duration of PN, median (IQR)	52 (21, 69.8)	38 (24.2–52.3)
DOL enteral feed started, median (IQR) (SD)	37.0 (27.8–65.8)	9.0 (2.5–17.5)
PN CH/kg/day, max, median (IQR)	15.0 (13.1–16.5)	14.0 (12.5–15.6)
PN lip/kg/day, max, median(IQR)	3.0 (2.3–3.0)	3.1 (3.0–3.5)
PN aa/kg/day, max, median (IQR)	4.0 (3.6–4.0)	3.5 (3.5–3.8)
Day start OV while on PN mean (SD)	18 ± 12.2	NA
Duration OV, median (IQR)	21.0 (13.5–97.0)	NA
Conj bili when OV started, (*μ*mol/L), mean (SD)	48.3 ± 16.60	NA

PN: parenteral nutrition, IUGR: intrauterine growth restriction, conj bili: conjugated bilirubin, CH: carbohydrates, lip: lipids, aa: amino acids, OV: Omegaven, DOL: days of life.

**Table 4:** Probability of cholestasis predicted by logistic regression model including duration of TPN and dose of carbohydrate.

Dose of CH (g/kg/day)	Duration of TPN (weeks)						
	2–4	4–6	6–8	8–10	10–12	12–14	14–16
5–10	2%	3%	6%	12%	20%	33%	48%
10–15	10%	17%	29%	43%	60%	74%	85%
15–20	39%	55%	70%	82%	90%	94%	97%
